# Smart technology and multibody simulation for skier safety: a structured narrative review and research roadmap

**DOI:** 10.3389/fspor.2026.1887357

**Published:** 2026-09-09

**Authors:** Giovanni Gerardo Muscolo, Daniele Binosi

**Affiliations:** 1 European Centre for Theoretical Studies in Nuclear Physics and Related Areas (ECT*), Fondazione Bruno Kessler (FBK), Trento, Italy; 2 Department of Physics, University of Trento, Trento, Italy

**Keywords:** alpine skiing, biomechanical modelling, digital twin, injury prevention, multibody simulation, skier safety, smart binding, wearable sensing

## Abstract

This structured narrative review examines how smart equipment, wearable and environmental sensing, digital twins, and multibody simulation can contribute to skier safety and prevention of ski-injury. The Scopus® search produced 58,919 aggregate query returns; after excluding two deliberately broad searches, 1,285 targeted-query records were screened and 74 sources were retained. The backward and the forward search for citation searches supported by Google Scholar™ identified another 76 sources, giving 150 included sources. Because the literature is heterogeneous in study design, outcome, and technological maturity, the evidence was synthesised qualitatively rather than by meta-analysis. The review distinguishes technologies that are already useful for measurement or protection from approaches that remain experimental or conceptual. It also identifies validation, calibration, uncertainty quantification, interoperability, false-alarm control, and field robustness as the principal barriers to real-time predictive safety systems. Finally, a non-validated Human–Skills–Equipment–Environment framework and a conceptual Skier Safety Index are presented solely as hypotheses for future research. The resulting roadmap prioritises prospective validation with human-subject data, standardised multimodal data sets, transparent model verification, and controlled evaluation of adaptive safety interventions.

## 1 Introduction

### 1.1 Skier performance, falls, and injury risk

Falls and injuries in alpine skiing arise from interactions among skier characteristics, technique, equipment, course design, speed, and snow conditions. In a study of 1,174 recreational skiers, Ruedl et al. (1) reported that 13.5% fell during the observation day and identified associations between fall occurrence and age, physical condition, ski geometry, speed, and snow conditions. These associations should not be interpreted as proof that any single factor causes a fall.

Training and education may also influence balance and injury risk. Zgurovski and Zdravcheva (2) reported an improved balance-related results with a training method that reduced vertical motion, while Zadic et al. (3) described improvements after a ten-week programme for young skiers. In contrast, short-term exercise-induced fatigue has been associated with impaired balance control, and fatigue has also been studied together with ski waist width in relation to knee-joint stability and balance (4, 5). External stimuli can also modify behaviour: Garner et al. (6) found an association between head-up-display use and skiing speed that varied with skill level. Together, these studies indicate that skier safety cannot be reduced to one variable or a technological device.

### 1.2 Scope, contribution, and article type

Existing reviews have provided important but largely domain-specific syntheses. Injury epidemiology and prevention have been reviewed from sports-medicine and racing perspectives (7, 8); ACL injury and reinjury have been treated in a dedicated narrative review (9); binding and ski–boot–binding safety have been considered in equipment-focused reviews (10, 11); terrain and kinematic data acquisition have been reviewed separately (12); and skiing robotics has recently been reviewed as its own engineering field (13). Consequently, smart equipment and sensing, computational biomechanics, and robotics are still distributed across partly separate review traditions, which makes it difficult to compare their evidential support, practical readiness, and validation requirements on a common basis.

The present article addresses this fragmentation gap as a *structured narrative review* with an engineering focus. It synthesises four bodies of literature that previous reviews have not jointly compared within one skier-safety framework: (i) smart skis, boots, bindings, and protective equipment; (ii) wearable and environmental sensing; (iii) digital-twin and multibody-simulation methods; and (iv) robotic and machine-learning approaches. The central contribution is therefore not another catalogue of technologies, but a cross-domain comparison of what is currently supported by evidence, what is measurement-ready, what remains experimental, and what validation is still required before safety-critical use.

The manuscript deliberately separates literature-derived evidence from the authors’ conceptual synthesis. Sections 2–5 review the published work. Section 6 critically compares technological readiness, implementation barriers, and validation needs and proposes a prioritised research roadmap. Section 7 contains an explicitly hypothetical integration framework. It does not present original experimental or simulation data and must not be interpreted as a validated safety-prediction method.

### 1.3 Methodology

The search covered English-language sources available up to July 2025. Scopus® was used for the structured database search, and Google Scholar™ was used for backward and forward citations because the topic spans biomechanics, sports medicine, engineering, robotics, standards and older conference literature that is not uniformly indexed in a single database. The complete set comprised 150 sources: 74 retained from Scopus® and 76 added through citation chaining and Google Scholar™.

The Scopus® search strings are reported in Table 1. The values in the table are *query-level returns*; the same record could be returned by more than one query, so the rows are not additive counts of unique studies. The deliberately broad searches “Falls” and “Fall AND Sport” were used only to gauge the size of the general literature and were excluded from detailed screening because of their low specificity. The remaining targeted queries yielded 1,285 records before cross-query de-duplication and screening.

**Table 1:** Scopus® search strings and query-level returns up to July 2025.

Search string	All years	2015–2025	2020–2025	Retained at query level
Falls	56,828	32,598	18,873	–
Fall AND Sport	806	377	190	–
Release AND Ski AND Binding	51	8	4	3
Collision AND Skiing	15	8	8	0
Alpine AND Skiing AND Binding	41	7	5	0
Alpine AND Skiing AND Boot	28	13	7	0
Alpine AND Skiing AND Release	20	3	2	0
Fall AND Ski	30	12	5	3
Boot AND Binding	31	9	5	5
Ski AND Safety	270	68	31	6
Boot AND Ski	92	32	15	9
Boot AND Skiing	61	20	10	0
Airbag AND Skiing	2	2	1	0
Impact AND Ski	106	48	32	0
Skiing AND Safety	323	90	44	7
Fall AND Skiing	116	46	27	0
Mechatronic AND Ski Binding	2	2	2	2
Robotic AND Ski Binding	0	0	0	0
Robotics AND Alpine AND Skiing	1	1	1	1
Robot AND Ski	18	9	5	12
Bionics AND Ski	2	2	2	1
Technology AND Alpine AND Skiing	10	10	8	6
Cyber AND Ski	1	1	1	1
Fall AND Prevention AND Alpine AND Skiing	4	1	1	1
Fall AND Prevention AND Ski	7	2	1	1
Multibody AND Alpine AND Skiing	1	1	1	1
Multibody AND Ski	4	1	0	2
Simulation AND Ski AND Fall	3	1	0	2
Balance AND Ski	22	14	10	8
Digital Twin AND Ski Injury	0	0	0	0
Digital Twin AND Snowfall	0	0	0	0
Digital Twin AND Ski	1	1	1	1
Digital Twin AND Alpine Skiing	1	1	1	1
Digital Twin AND Skiing	1	1	1	1
Snowfall AND Alpine Ski	21	0	0	0
Aggregate query returns	58,919	33,389	19,294	–
Targeted-query returns after removing the two broad searches	1,285	414	231	–
Unique Scopus sources included after de-duplication and screening	–	–	–	74

Counts can overlap across rows because one paper may be returned by several queries. The final Scopus® set contained 74 unique sources after cross-query de-duplication and screening.

Sources were eligible when they: (i) concerned alpine skiing or an adjacent biomechanical or engineering domain with a clearly stated application to skier safety; (ii) addressed injury mechanisms, equipment, sensing, course/environmental factors, digital twins, multibody or musculoskeletal modelling or robotic test systems; (iii) provided sufficient methodological or technical detail to support interpretation; and (iv) were available in English. Exclusion criteria were duplicate records, topics without a plausible connection to alpine skiing, purely promotional or commercial descriptions, insufficient methodological detail, and unavailable full text. Conference papers and preprints were retained only when they described a technically relevant method and no equivalent journal article was identified.

**Figure 1:**
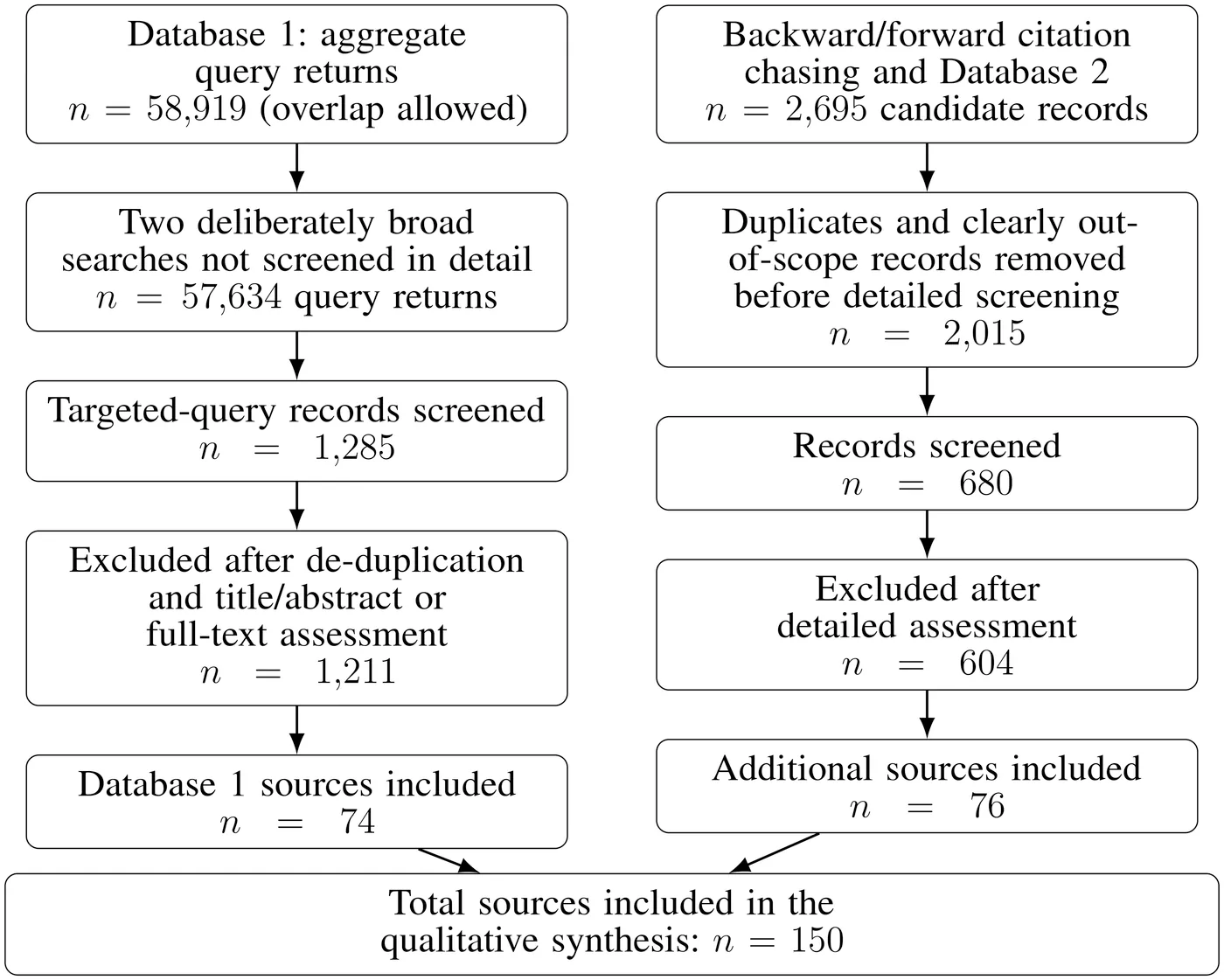
Flow diagram of the two-stage search and screening process databases: (1) Scopus®; (2) Google Scholar™. Exclusion reasons included duplication, lack of relevance to alpine skiing or skier safety, insufficient methodological detail, and unavailable full text. Query-return counts are not unique-record counts.

The first author performed title/abstract screening and full-text assessment. The final set and the thematic synthesis were reviewed by both authors. No formal risk-of-bias instrument was applied because the included sources ranged from epidemiological studies and standards to sensor-validation studies, numerical models, and engineering prototypes. For the same reason, no meta-analysis was attempted. The evidence and readiness assessments in Section 6 are therefore qualitative and should not be interpreted as formal grade ratings or the certification technology-readiness-level. Figure 1 provides a flow-diagram description of the selection process (14).

## 2 Epidemiology and mechanisms of ski injuries

Koehle et al. (7) reviewed alpine-ski injuries and prevention studies published between 1989 and 2002. The historical decline from approximately 5–8 injuries per 1,000 skier-days in the 1970s to approximately 2–3 per 1,000 skier-days from the 1990s onward coincided with major changes in boots and bindings (7). This temporal association supports the relevance of equipment design, but it does not establish the contribution of each individual design change itself. Binding-release behaviour has also been associated with lower-leg and knee injury patterns (10, 15).

The knee is the lower-body joint most frequently involved in snow-sports injuries (16, 17). Large external loads arise from the skier–equipment–snow interaction and can be amplified by the long lever arm of the ski. Hardware-in-the-loop systems with an artificial knee have therefore been developed to reproduce forces and moments under controlled laboratory conditions (18). Ski geometry, including sidecut radius, is associated with turning mechanics and loading patterns relevant to severe knee injury (19); the available evidence should be interpreted as a biomechanical and epidemiological association rather than as a simple one-variable causal relationship.

After injury, return-to-sport protocols must restore biomechanical and perceptual-motor performance, while reinjury remains a concern (9, 20). In a survey of 541 patients, Shea et al. (21) classified the reported knee-injury mechanisms such as valgus-external rotation (32%), phantom foot (25%), hyperextension (19%), boot induced (7.8%), collision (2.2%), and other mechanisms (15.6%). Course geometry, turning mechanics, and speed are potentially modifiable at the competition or policy level, although many contextual constraints remain (22).

Injury mechanisms also differ by discipline: Super-G and Downhill involve higher speeds and more jumps, whereas Giant Slalom produces high loads during repeated turns (23). Forward and backward twisting falls are prominent mechanisms in female skiers with ACL injury (24). The Abbreviated Injury Scale (AIS), used in trauma research, ranks the severity of anatomical injury from 1 (minor) to 6 (maximal/currently untreatable); it is a severity scale, not a measure of injury incidence (25). These observations support a multidimensional safety perspective that distinguishes injury occurrence, injury mechanism, and injury severity.

## 3 Smart technology in snow equipment

### 3.1 Skis

Flexural and torsional ski deformation provide information about ski loading, edge angle, and the skier–equipment interaction (26). Recent sensing approaches include a batteryless piezoelectric system that measures ski deformation and vibration and transmits data to a smartphone (27), printed sensor arrays for torsional-deflection measurements (28, 29), and low-cost inertial measurement units (IMUs) for estimating ski inclination and acceleration (30). These studies support instrumented skis as measurement platforms, although their direct effect on injury reduction has not yet been demonstrated.

### 3.2 Ski boots

Ski boots transmit loads between the lower limb and the binding, so their geometry, mass, stiffness, closure, and alignment can affect both performance and injury-relevant mechanics (31). The proposed evaluation methods include standardised measurements of ankle range of motion (32), on-snow comparisons of commercial boot closures (33), and laboratory protocols for flexural behaviour under realistic loading (34, 35). Boot range of motion (36), viscoelasticity and its dependence on angular velocity (37), and structural stiffness (38, 39) are therefore important parameters for characterisation.

Embedded IMUs have also been used to quantify skiing technique and distinguish motion quality in skiers of different skill levels (40), while instrumented boots can support automatic turn detection (41). Alignment interventions such as canting wedges have been associated with changes in kinematics, kinetics, and postural-control measures (42). Collectively, these studies show that boots can serve both as mechanical components and as sensor platforms; however, evidence that a specific smart-boot configuration prevents injuries remains limited.

### 3.3 Ski bindings and release

#### 3.3.1 History and techniques

**Figure 2:**
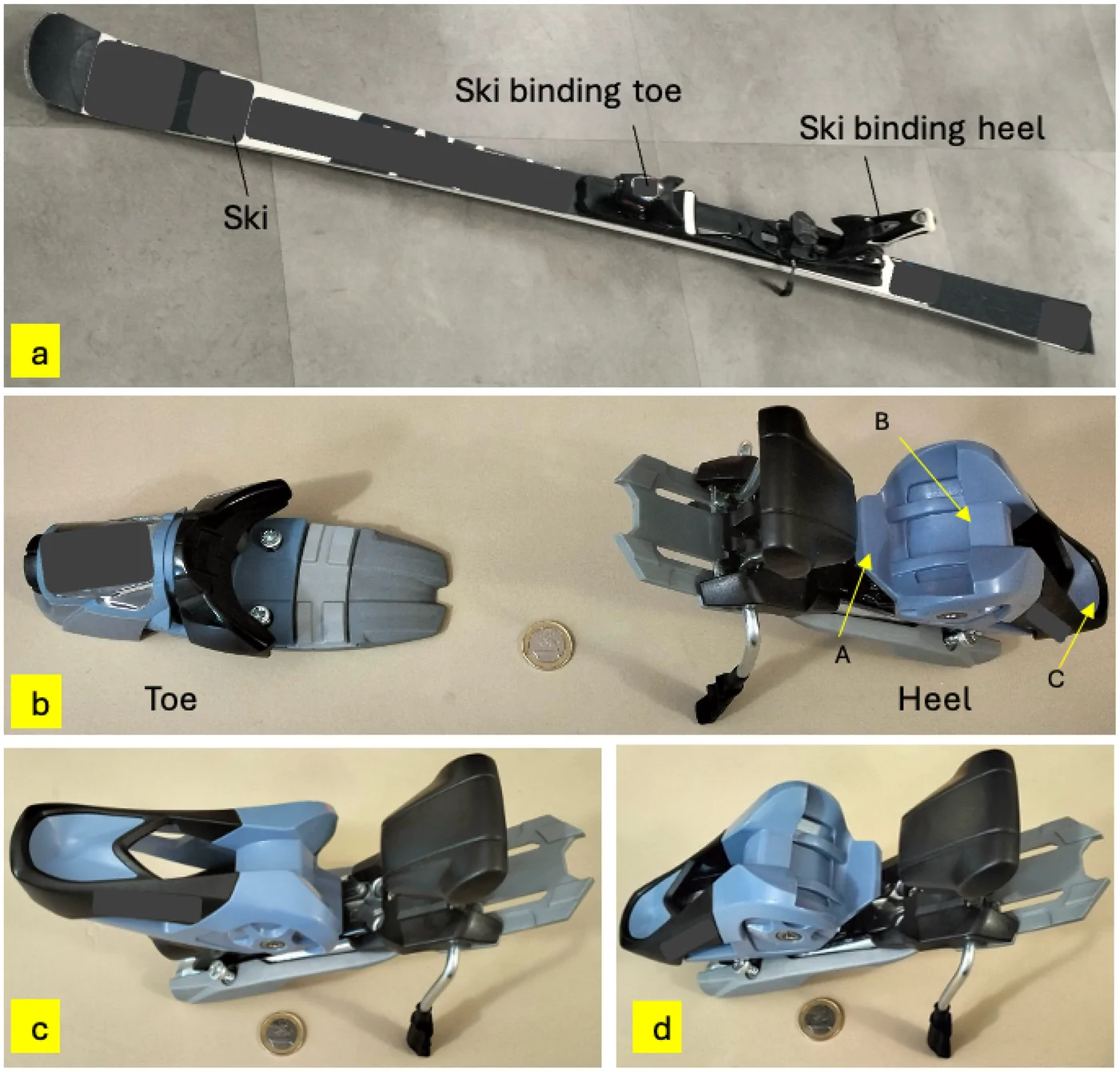
Alpine ski and binding components: **(a)** Ski with toe and heel units; **(b)** Toe and heel units shown separately: on the heel unit, A indicates the surface pressed during step-in/closure, B the boot-heel retention/contact surface, and C the manual release lever; **(c)** Heel unit in the locked configuration; **(d)** Heel unit in the released configuration.

In alpine skiing, a conventional binding consists of a toe unit and a heel unit attached to the ski. The ski boot is retained between these two units; the boot itself is not shown in Figure 2, which is included to identify the principal binding components and to illustrate the operating surfaces of the heel unit.

The American Society for Testing and Materials (ASTM International) specifies test methods to measure quasi-static release moments of alpine ski bindings (43). Such testing typically applies controlled loads through cables and force–torque transducers to assess release behaviour.

Release bindings emerged in the mid-twentieth century and generally use a spring-loaded mechanism that releases when an applied load exceeds a prescribed threshold. Their development and professional adjustment were associated with large reductions in tibial and ankle injuries, whereas knee injuries did not decline to the same extent (10, 15). Historical evidence also indicates that non-release is associated with greater injury risk than release of at least one ski. These findings span different binding generations and study designs, so they should be interpreted as population-level associations rather than as precise estimates for current products.

Research on active and electronically modulated release dates back several decades. Early studies examined binding dynamics and ski–boot relative motion under impact (44, 45); later prototypes used strain-gauge measurements, computer-controlled hydraulic actuation, electrically modulated twist release, heel release, and electromechanical mechanisms with mechanical support (46–49). These studies established the technical feasibility of sensing and active release, but also exposed the difficulty of achieving reliable multidirectional release without unacceptable inadvertent activation. Therefore appropriate calibration and professional adjustment remain essential (10).

Additional measurements at the boot–ski interface can improve estimates of the loads transmitted to the tibia (50). Such estimates may inform binding design and calibration, but they cannot yet guarantee injury avoidance because tissue loading, muscular response, fall dynamics, and release timing interact in complex ways.

#### 3.3.2 Smart bindings

Because conventional release settings provide limited protection against several knee-injury mechanisms, mechatronic bindings have been investigated as a possible extension of passive systems. Robotic test rigs can apply multi-axis loads to study binding release and the resulting knee-relevant reactions (51). The principal design problem is to distinguish hazardous loading from normal skiing while avoiding both missed releases and inadvertent releases. Low-speed falls, muscle activation, release timing, and multi-axis loading therefore require particular attention.

Technical reviews have proposed velocity-dependent logic and other active strategies to improve sensitivity to knee-relevant loading (11). Epidemiological studies also report sex-related differences in binding-release behaviour and suggest that adherence to ISO 11088 settings can improve self-release outcomes (52, 53). In one cohort of 1,369 injured recreational skiers, 39% reported that the binding had not released during the injury event (53); this association does not establish that non-release caused every injury.

A proposed mechatronic system would require, at minimum, information on knee angle, quadriceps and hamstring activation, loads at the feet or centre of mass, ski velocity, and skier-specific characteristics (54). These inputs could be obtained from IMUs or stretch sensors, electromyography (EMG), pressure or force–torque sensors, and positioning systems. Although this sensor set is technically plausible, clinical effectiveness, false-release performance, fail-safe behaviour, power supply, certification, and maintenance remain unresolved.

## 4 Smart technologies and sensing modalities in the snow environment

### 4.1 Smart technologies and sensing modalities

Reports of serious falls and injuries have contributed to renewed safety measures by the International Ski and Snowboard Federation (FIS), including mandatory airbag use in speed disciplines (55–57). At ski-area and competition level, safety systems include course design, barriers, protective equipment, and monitoring technologies (58). Body-worn IMUs, pressure sensors, and other instruments can quantify acceleration, posture, vibration, and interface loading, thereby supporting exposure assessment and biomechanical analysis (8, 59, 60). Course geometry, terrain, energy, speed, and skiing technique are additional variables relevant to injury mechanisms (12, 61, 62).

Safety barriers have been investigated through full-scale impact tests with instrumented anthropomorphic dummies and through finite-element analysis of safety nets (63, 64). These studies illustrate the role of controlled physical and numerical testing in assessing impact mitigation. In Super-G and Giant Slalom, where speeds commonly reach approximately 60–80 km/h, safety-oriented design and monitoring may target wearable and embedded sensing, gate design and placement, impact-attenuation systems along the course, helmets and other protective equipment, and fatigue or workload monitoring. These are cross-cutting safety targets rather than separate sensing technologies, so they are summarised here instead of being treated as additional technical subsections.

Carving turns are prominent in injury-video analyses, and their mechanics can be studied using complementary modalities such as video, pressure insoles, instrumented boots, and IMUs (65). Here, a *sensing modality* denotes the physical quantity and measurement principle used by an engineered system: for example, vision, inertial sensing, pressure, force, positioning, or physiological monitoring.

Table 2 groups representative technologies by sensing modality and output. Machine learning is listed separately because it is an inference method applied to sensor data rather than a physical sensor.

**Table 2:** Smart technologies, corresponding sensing modalities, typical outputs, and representative sources used in alpine skiing.

Technology	Sensing modality	Analysis type	Typical output	Representative source
Visual camera (VC)	Vision	Kinematics	Pose and trajectory	(66)
Global navigation satellite system (GNSS)	Navigation	Kinematics	Position and speed	(67)
Inertial measurement unit (IMU)	Inertial	Kinematics; dynamics	Orientation and acceleration	(68)
Force or pressure measurement	Mechanical	Statics; dynamics	Interface load; estimated GRF	(69)
Health sensor (HS)	Physiological	Biomechanics; physiology	Heart rate; fatigue proxies; muscle activity	(70)
Machine learning (ML)	Data inference	Classification; regression	Event detection; reconstruction; decision support	(71)

#### 4.1.1 Visual cameras and machine learning

Video-based reconstruction provides body pose, trajectory, and event timing without placing equipment directly on the skier. It has been used for retrospective analysis of World Cup injuries (66, 72). Among 69 analysed injury cases, 55 occurred during turning and 13 during jumping; knee injuries were observed predominantly during the skiing phase, whereas head and trunk injuries were observed mainly after falls or impacts (66). These frequencies describe the analysed cases and should not be interpreted as population-wide causal probabilities.

Multi-camera systems, including combinations of fixed and drone-mounted cameras, can reconstruct skier trajectories (73). Their performance is limited by occlusion, snow spray, weather, viewing geometry, and complex body poses. Machine-learning and computer-vision methods can improve keypoint detection and reconstruction in partially occluded sequences (71), but their accuracy must be evaluated on independent data and under representative field conditions. Video can also be combined with EMG to examine muscle responses associated with specific injury mechanisms (74).

#### 4.1.2 Global navigation satellite systems

Global navigation satellite systems (GNSS), particularly differential GNSS, are used to estimate skier position, velocity, trajectory, and external-force-related quantities (67, 75). Competitive-skiing studies have compared kinematic and impact-related parameters between disciplines and group athletes (76). Such findings are observational and depend on the course, sample, and measurement protocol. GNSS provides valuable global motion information, but terrain, satellite visibility, antenna placement, and sampling or synchronisation errors constrain its use as a stand-alone safety sensor.

#### 4.1.3 Inertial and physiological sensing

IMUs can estimate segment orientation, joint angles, acceleration and vibration, and can be combined with GNSS or video to improve motion reconstruction (68, 77, 78). Their principal limitations are sensor bias, integration drift, magnetic disturbance, soft-tissue or garment motion, and mounting variability. Therefore, functional calibration and drift-correction procedures are essential (68, 78).

Physiological detection can add information on heart rate, muscle activity, and fatigue-related state (70, 79–84). EMG is particularly relevant to muscle activation and co-contraction, but it does not directly measure muscle force and is sensitive to electrode placement, motion artefact, clothing, temperature, and signal normalisation. Although many health-sensor technologies are mature in clinical or daily monitoring, robust deployment during competition remains challenging.

#### 4.1.4 Ground-reaction-force reconstruction

Ground reaction forces (GRFs) are central to the ski–snow interaction and to estimates of joint loading, yet they are difficult to measure accurately during alpine skiing. Instrumented ski–binding interfaces with force–torque cells have been validated against external vision systems and tested during snow turns (69, 85). Foot positioning and equipment setup affect measured kinematics and kinetics (86). Turn-to-turn GRF asymmetries have also been observed, but muscular-strength asymmetry alone did not explain them in elite slalom skiers (87).

Portable force platforms and pressure insoles provide different information. Pressure insoles are lighter and less intrusive, whereas force platforms measure resultant external loads more directly. Comparisons show that the methods are not interchangeable and that pressure insoles can underestimate GRFs (88, 89). Differential GNSS has also been used to estimate external forces in speed disciplines (90), and strain-gauge transducers have long been used at the ski–boot interface (91, 92). These methods support biomechanical measurement; their use for real-time injury prediction requires additional validation, synchronisation, and uncertainty analysis.

### 4.2 Robotics in alpine skiing

Robotic skiing systems provide controlled test beds for balance, perception, turning, obstacle avoidance, and ski–snow interaction. A recent systematic review identified a small set of technically relevant studies within a much larger literature (13). The main challenges are real-time terrain perception, robust balance control, low-friction and deformable contact actuation, and transfer from simulated to real snow (13, 93, 94).

Obstacle-avoidance and turning controllers have used ski angle, visual perception, zero-moment-point concepts, and deep reinforcement learning (95–98). Other systems have explored passive turning, autonomous navigation, and vision-only balance control (99–104). Instrumented anthropomorphic mannequins and robotic test rigs can also reproduce impacts or loading scenarios that would be unsafe or difficult to study with humans (105).

These platforms are valuable for repeatable engineering experiments, but current ski robots do not demonstrate that robotic intervention reduces human injury. Their near-term contribution is more plausibly as a test infrastructure for sensors, contact models, protective systems, and control algorithms than as autonomous safety devices deployed alongside skiers.

## 5 Digital twin and multibody simulation of skiers

### 5.1 General overview

A digital twin (DT) is more than a static virtual replica: it is a computational representation that is updated using data from its physical counterpart. In this review, the term DT is reserved for data-connected models, whereas multibody simulation (MBS) denotes a numerical mechanical model that can be embedded in a multiphysics environment. A skiing-related DT has, for example, been used to analyse environmental wind effects (106). Real ski simulators can reproduce selected external conditions for training and kinematic studies (107, 108), while MBS can represent linked body segments, skis, contact, and aerodynamic effects. During slalom, the ski sidecut and the angle of the edge constrain the trajectory of a carved turn (109).

Simulating skier impacts with obstacles can support safety analyses. As highlighted by Dorsemaine et al. (110), the head and trunk are critical body regions to protect.

Computational studies become more informative when their outputs are compared rather than only their model architectures. In the flexible-ski MBS of Eberle et al. (111), measured random slope topography was propagated through an Euler–Bernoulli ski model; the simulations showed that slope irregularities contributed substantially to ski-shovel vibration, indicating that idealised smooth-slope contact can omit an important excitation mechanism. Aerodynamic modelling provides a complementary example: Meyer et al. (112) reported errors of approximately 11.00%–14.28% for generalised drag models and 4.52%–5.30% for individualised models, and the field application predicted about 10% greater aerodynamic energy loss for a dynamic than for a compact technique. These results show both the value of person-specific modelling and the fact that performance-relevant losses cannot be inferred from posture-independent coefficients alone. Earlier reduced-order work also estimated kinetic friction and drag area from downhill motion (113).

To reduce fall and injury risk in alpine skiing, knee-angle measurements could provide useful input for ski-binding release systems. In Klous et al. (114), joint load and injury statistics are studied and the knee appears to be more heavily loaded than the ankle in the reported pilot data. In Hermann et al. (115), two sensors (piezoresistive and capacitive) were compared to measure knee angle.

### 5.2 Ski–snow physical interaction

Ski–snow interaction is fundamental to alpine-ski mechanics (116, 117). Friction depends on temperature, speed, load, base material, snow microstructure, lubrication, capillarity, and meltwater formation, making a single transferable coefficient difficult to define (116). During carving, snow is compressed, penetrated, and sheared; hypoplastic contact laws can represent some of this behaviour more realistically than simple elastic penetration models (118–120).

Experimental and numerical approaches include measurements of snow resistance after ski penetration (121), finite-element models of ski–snow contact (122), coupled ski and elastic-foundation models (123), and studies of polyethylene friction under different loads and temperatures (124). Contact-pressure models have represented the ski as a variable-section Euler–Bernoulli beam and the snow reaction through penetration and shear components (125). A free-gliding tribometer reported kinetic-friction coefficients of approximately 0.02–0.0225 over a speed range of 1–5.6 $m\;s^{-1}$ under the tested conditions (126). Evidence of frictional meltwater further illustrates the dependence of contact behaviour on thermal processes (127).

Because contact parameters vary with snow state and experimental protocol, model calibration should report the tested operating range and propagate uncertainty into predicted skier loads.

The available simulations also show that contact-model detail changes the conclusions that can be drawn. The finite-element model of Federolf et al. (122) reproduced experimental snow-penetration behaviour and, after including the groove formed by the front of the ski, could reproduce instantaneous turn radii observed in carved turns; however, the groove formulation also introduced substantial numerical instability. In the quasi-static contact-pressure study of Heinrich et al. (125), increasing edge angle and distributing load across both skis increased resistance to shearing. Thus, adding contact realism can improve agreement with measured turn mechanics, but may reduce numerical robustness and increase parameter dependence.

### 5.3 Human-like motion and multibody models in alpine skiing

Models of alpine skiing range from idealised analytical descriptions to detailed musculoskeletal simulations. The ideal carving equation provides a simplified relation among geometry, inclination, and turning (128). A two-dimensional five-link model represented the arm, trunk, thigh, shank, and boot, with distributed ski contact and aerodynamic drag, but without an active control system (129). Other reduced-order approaches have treated the skier as a point mass for trajectory optimisation (130), while inertial and force sensors have supported dynamic analyses of ski turns (131).

More detailed models include a planar musculoskeletal skier with flexible segmented skis (132), analytical centre-of-mass models for carved turns (133), and rigid-body optimal-control formulations for trajectory and posture (134, 135). Related musculoskeletal studies in sprinting demonstrate how data-tracking simulations can reproduce measured motion when the model, objective function, and constraints are appropriately selected (136). Komissarov developed a series of theoretical models for pure carving, side slipping, snow machining, wedge turns, and centrifugal-pendulum dynamics with a retractable leg (137–142). Energy-based indicators have also been proposed for performance evaluation (143).

These models differ markedly in dimensionality, contact representation, degrees of freedom, control assumptions, and intended output. Reduced models are useful for interpretation and optimisation, whereas detailed models can estimate joint or tissue loading but require substantially more data and validation.

Greater model complexity is not automatically advantageous for every output. Using data from nine skiers, Hladnik et al. (143) found no significant difference between a mass-point representation and a 15-segment linked-body model for the specific mechanical-energy metric used to evaluate performance; the additional segmental and rotational energies were negligible for that purpose. By contrast, injury-related quantities such as joint moments or ligament loads require anatomical detail that a point-mass model cannot provide. Model complexity should therefore be selected from the target output and validation question rather than treated as an end in itself.

### 5.4 Multibody simulation

Multibody and musculoskeletal models are widely used to study tendon elasticity, muscle coordination, curved running, and lumbar-spine biomechanics (144–146). Their outputs may include kinematics, GRFs, joint moments, joint reaction forces (JRFs), muscle forces, and injury-relevant tissue loads. Such estimates can support offline analysis and hypothesis testing, but their reliability depends on model structure, input data, parameter identification, and validation.

The ski–snow interaction remains a major source of uncertainty: errors in contact geometry, friction, ski flexibility, scaling, muscle representation, and control can propagate into predicted joint loads. Impact-reconstruction models for skiing and snowboarding (147), and related optimal-control models for surfing (148), illustrate the broader potential of computational biomechanics while also showing that transfer between sports requires explicit validation.

Rather than cataloguing software, Table 3 compares representative computational studies by data connection, quantitative finding, and principal interpretive limitation. Studies have used both reduced-order and detailed musculoskeletal models (129, 132, 149–151), with validation or data tracking ranging from mechanical experiments to human kinematics, GNSS, force, pressure, and EMG measurements (146, 152–154).

**Table 3:** Critical comparison of representative computational-biomechanics studies relevant to alpine skiing.

Study/model	Data connection or validation	Representative result	Interpretation and limitation
Flexible-ski MBS (111)	Random slope surfaces derived from high-resolution laser scanning	Random slope irregularities contributed substantially to simulated ski-shovel vibration.	Shows the importance of spatially variable contact excitation; study considered planar straight schussing rather than turning or tissue loads.
Aerodynamic model (112)	Wind-tunnel data from 15 skiers plus a field application	Generalised models: 11.00%–14.28% error; individualised models: 4.52%–5.30%; dynamic technique produced about 10% more aerodynamic energy loss than compact technique.	Demonstrates benefit of individualisation for aerodynamic performance; not an injury model and static wind-tunnel postures approximate a dynamic turn.
Finite-element ski–snow model (122)	Snow-penetration experiments and measured instantaneous turn radii	Including the snow groove enabled reproduction of measured turn radii, but introduced substantial numerical instability.	Improved contact realism can improve mechanical agreement while increasing numerical sensitivity.
Perturbed landing model (149)	Baseline simulation reproducing a measured landing; 1,000 perturbed simulations	Trunk orientation was the strongest predictor and accounted for about 60% of the variance in simulated peak ACL force.	Useful mechanistic sensitivity result, but derived from a two-dimensional model and simulated perturbations rather than prospective injury outcomes.
3D turning model (153)	Professional-skier kinematics tracked by a 53-DoF, 94-muscle model	Joint-angle RMSD 0.50${}^{\circ }$–2.72${}^{\circ }$; speed RMSD 0.12 m/s; about 60% of total GRF acted on the outside leg; peak lumbar-extension and outside-knee-extension moments were 1.88 and 1.02 N m/kg, respectively.	Strong kinematic tracking for one turning manoeuvre; single-skier data and omitted ski torsion limit external validity.
Muscle/ACL turning model (154)	3D musculoskeletal tracking of experimental skier data with sensitivity analyses	Peak ACL force was 211 N on the outside leg and 15 N on the inside leg; sensitivity analyses placed the outside-leg peak between 170 and 244 N.	Provides tissue-load estimates for a balanced turn, but ACL force remains model-derived and depends on knee and muscle-model assumptions.
Mass-point vs. 15-segment model (143)	GNSS and inertial whole-body data from nine skiers	No significant difference was found between models for the analysed specific mechanical-energy performance metric.	Shows that higher complexity is not always required for performance metrics; the conclusion does not extend to joint or ligament loading.

Numerical values are study-specific and should not be generalised beyond the reported task, participant(s), and model assumptions.

Across these studies, the strongest quantitative agreement is currently reported for observable kinematics, trajectory, speed, or mechanically testable contact behaviour. Joint moments, muscle forces, and ACL forces are increasingly data-constrained but remain model-derived quantities. In particular, Heinrich et al. (153) tracked measured joint angles within 0.50${}^{\circ }$–2.72${}^{\circ }$ and speed within 0.12 m/s before estimating joint moments, whereas Heinrich et al. (154) subsequently estimated an outside-leg peak ACL force of 211 N in a balanced turn. By contrast, the landing simulations of Heinrich et al. (149) explored injury-prone perturbations and found trunk orientation to explain about 60% of the variance in simulated peak ACL force. These are complementary levels of evidence: tracking accuracy supports model fidelity for measured observables, while injury-relevant tissue loads still require independent validation before they can support prediction or intervention.

### 5.5 Model validation and transferability

Predictive multibody models require validation at several levels. OpenSim provides a transparent framework for model scaling, inverse kinematics, inverse dynamics, muscle analysis, and forward simulation, but the numerical output remains conditional on the model structure and the quality of the input data (155, 156). Validation should therefore compare model outputs with independent measurements rather than with the same data used to drive or calibrate the model. Relevant comparisons include joint kinematics against optical motion capture, external loads against instrumented bindings or force platforms, muscle activity against electromyography, and predicted injury-relevant loads against laboratory or field measurements.

Lower-limb asymmetry is an instructive example. Alpine-skiing data show that turn-to-turn GRF asymmetries cannot be inferred directly from strength asymmetry alone (87). Computational studies of unilateral weakness demonstrate how controlled asymmetry can be introduced into a musculoskeletal model, but also emphasise the need for human-subject validation and uncertainty analysis before transfer to safety decisions (157). For skier digital twins, left–right asymmetry, fatigue, prior injury, and equipment setup should therefore be treated as person-specific parameters to be estimated and validated, not as assumed constants.

## 6 Critical synthesis and research roadmap

### 6.1 Evidence strength and practical readiness

The reviewed technologies differ substantially in evidential support and readiness. Table 4 uses four qualitative categories: *established* (routine or standardised use with direct safety relevance), *measurement-ready* (validated for measurement but not for injury reduction), *experimental* (prototype or laboratory evidence), and *conceptual* (mainly simulation or proposed future use). These categories are a narrative synthesis, not a formal regulatory or technology-readiness assessment.

**Table 4:** Qualitative comparison of evidence and practical readiness.

Technology family	Readiness for safety use	Evidence supporting current use	Principal limitation	Representative sources
Conventional mechanical bindings	Established for calibrated release and reduction of some lower-leg injuries	Standards, epidemiology, and long-term equipment experience	Limited protection against complex knee-loading mechanisms; setting and maintenance errors	(10, 15, 43)
Smart or mechatronic bindings	Experimental	Laboratory prototypes, biomechanical models, and technical reviews	False release, multi-axis sensing, decision thresholds, certification, power, and fail-safe design	(11, 46–49, 54)
IMU, GNSS, video, and instrumented-ski systems	Measurement-ready	Validation against video, optical systems, or differential GNSS; retrospective injury analysis	Drift, occlusion, calibration, latency, and lack of prospective injury-prediction validation	(28–30, 66, 72, 109, 131)
Force platforms and instrumented interfaces	Measurement-ready in research; limited field deployment	Direct load measurement and comparison studies	Added mass and volume, durability, calibration, and pressure-insole underestimation of GRF	(69, 85, 88, 89)
Airbags and protective systems	Deployed for impact mitigation; effectiveness remains context dependent	Policy adoption, impact studies, and injury-mechanism evidence	Trigger timing, body-region coverage, false deployment, comfort, and discipline-specific evidence	(55–57, 63, 64, 66, 110)
Digital twins and MBS	Experimental for skier safety	Published mechanical and musculoskeletal simulations	Contact modelling, personalisation, uncertainty, computational cost, and external validation	(111, 132, 149, 152–154)
Machine learning and robotics	Conceptual to experimental	Tracking algorithms, robot prototypes, and controlled demonstrations	Generalisation, explainability, field safety, latency, and limited evidence of human injury reduction	(13, 71, 95, 97, 98)

“Readiness” refers to the specific safety application, not merely the availability of the underlying hardware.

The strongest present-day contribution of sensing technologies is therefore *measurement*, not demonstrated injury prevention. Conventional bindings and protective equipment already have direct safety functions, although their protection is mechanism-specific. Smart bindings, predictive digital twins, robotic intervention, and AI-guided real-time decisions remain research targets. Presenting these approaches as equally mature would overstate the evidence.

### 6.2 Limitations of the review and qualitative assessment

This study is a structured narrative review rather than a systematic review or meta-analysis, and only English-language sources were considered. The evidence base spans heterogeneous designs, including epidemiological studies, standards, sensor-validation studies, numerical simulations, and engineering prototypes. Consequently, the readiness categories in Table 4 were assigned qualitatively by the authors through discussion and consensus rather than by blinded independent scoring or a validated technology-readiness instrument. The categories should therefore be interpreted as an organising synthesis rather than as reproducible ratings. Citation chaining and the broad search strategy reduce, but cannot eliminate, the possibility of missed studies, source-selection bias, or author judgement influencing the synthesis.

### 6.3 Technical and operational limitations in real-world skiing

Laboratory performance does not automatically transfer to alpine skiing. Snow, moisture, low temperature, impacts, vibration, changing illumination, clothing, and repeated mounting can affect sensors and communication systems. IMUs are subject to bias and drift; video is vulnerable to occlusion and limited camera geometry; GNSS performance varies with terrain and satellite visibility; pressure insoles can underestimate external loads; instrumented bindings add mass and may alter the boot–binding interface. Multimodal fusion can reduce some weaknesses, but it also creates new requirements for time synchronisation, calibration, missing-data handling, and uncertainty propagation.

Safety-critical actuation introduces additional constraints. An adaptive binding or airbag must operate with bounded latency and a demonstrably acceptable balance between missed events and false activation. A false binding release may itself cause a fall, while an overly conservative threshold may provide no additional protection. Any real-time alert must also avoid distracting the skier. Battery life, fail-safe behaviour, maintenance, interoperability, cybersecurity, data protection, and certification must be considered from the beginning rather than after algorithm development.

Variation across sex, age, skill, discipline, prior injury, boot geometry, binding settings, and snow conditions limits the validity of universal thresholds. Models and algorithms should report the population and operating envelope for which they were developed. Transfer to another discipline or skier group requires external validation.

### 6.4 Validation requirements for predictive safety systems

A staged validation strategy is required before sensor-model systems can be used for safety decisions:

1. **Sensor validation:** calibrate each sensor against an appropriate reference and quantify accuracy, repeatability, drift, temperature sensitivity, and mounting effects.
2. **Biomechanical validation:** compare predicted kinematics, GRFs, joint moments, muscle activity, and contact forces with independent human-subject measurements. Report subject-specific scaling and uncertainty.
3. **Model verification and sensitivity analysis:** test numerical convergence, contact-law assumptions, parameter identifiability, and sensitivity to friction, snow properties, joint stiffness, and measurement error.
4. **Prospective outcome validation:** evaluate fall or injury-risk predictions on data not used for development. Report discrimination, calibration, sensitivity, specificity, false-alarm rate, and decision latency rather than accuracy alone.
5. **Field and human-factors validation:** test robustness, comfort, behaviour change, usability, and failure modes in representative snow, weather, course, and competition conditions.

Only after these stages should an intervention such as adaptive release, an airbag trigger, or a real-time warning be evaluated in a controlled safety study. Simulation can reduce the experimental search space, but it cannot substitute for empirical validation.

### 6.5 Prioritised research gaps and roadmap

The literature suggests the following priorities, ordered by their ability to unlock several downstream applications:

1. **Standardised multimodal data sets.** Collect synchronised video, IMU, GNSS, instrumented-binding or force-platform, equipment, snow, and outcome data with common metadata and calibration protocols.
2. **Validated skier-specific models.** Develop digital twins and MBS that explicitly represent ski–snow contact, equipment settings, biomechanical inertial parameters, skier-snow crash impacts, fatigue, left–right asymmetry, and uncertainty; validate them out of sample.
3. **Common benchmarks.** Define shared tasks for turn segmentation, centre-of-mass (CoM) estimation, GRF reconstruction, joint-load prediction, fall detection, and pre-impact classification, with transparent train/test separation.
4. **Prospective evaluation of adaptive systems.** Test smart bindings, airbags, and warnings using predefined thresholds and report false release, false alarm, missed event, latency, and adverse-event metrics.
5. **External validity and implementation.** Evaluate systems across skier populations, disciplines, equipment configurations, and snow conditions, while addressing certification, privacy, maintainability, and user acceptance.

Bioinspired surface or traction concepts and ski robots may provide useful test beds (13, 158), but they should not displace the more immediate need for validated measurements and interoperable data. The near-term priority is not a fully autonomous “intelligent skier-safety system”; it is a trustworthy measurement and validation infrastructure on which such systems could eventually be built.

## 7 Conceptual integration framework

### 7.1 Status of this section

**Figure 3:**
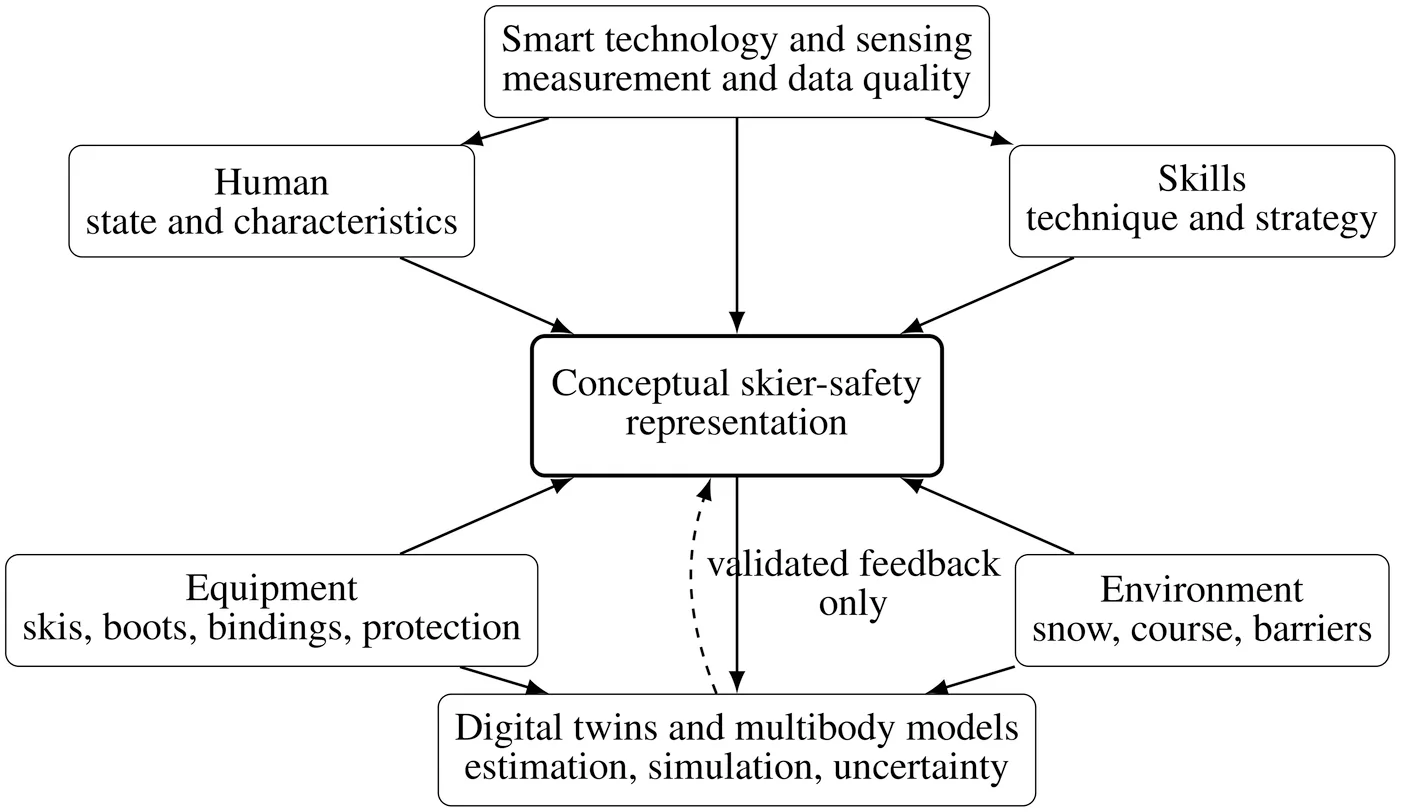
Conceptual integration of the four domains with sensing and modelling. Arrows indicate information flow, not validated causal relations. Any feedback from a model to a safety decision would require the validation stages described in Section 6.4.

The Human–Skills–Equipment–Environment framework shown in Figure 3 and the Skier Safety Index (SSI), shown in Equation 2 below are conceptual proposals derived from the reviewed literature. They are not validated, do not constitute clinical or operational decision tools, and do not contain new experimental or simulation results.

### 7.2 Rationale for the H–S–Eq–En domains

The four domains were selected to separate determinants that are often conflated: characteristics or states of the person (Human), learnt behaviour and task execution (Skills), artefacts worn or used by the skier (Equipment), and external course or snow conditions (Environment). Candidate variables were included when they met at least three of four criteria: (i) they were linked in the reviewed literature to falls, injury mechanisms, or injury severity; (ii) they can in principle be measured or estimated; (iii) they are potentially modifiable or actionable; and (iv) they add information not already represented by another domain.

The three-of-four rule is a non-validated heuristic introduced to make this conceptual selection process explicit; it is not an empirically derived threshold. The criteria were applied qualitatively by the two authors and resolved through discussion rather than by blinded independent scoring. No sensitivity analysis was performed to compare two-of-four, three-of-four, or four-of-four inclusion rules. The three-of-four choice was intended to require support on most criteria while allowing one criterion to be unmet, but a different threshold could change the variables retained. Accordingly, the variable set should be regarded as illustrative. Future work should formalise the criteria, quantify inter-rater agreement, and test threshold sensitivity before the framework is used analytically.

Table 5 lists illustrative variables and their evidence basis. The list is intentionally non-exhaustive. Age, sex, previous injury, risk-taking behaviour, visibility, snow temperature, equipment maintenance, and many other factors may be relevant and should be added or removed according to the population and use case. The framework is therefore a taxonomy for study design, not a claim that 15 variables are universally sufficient.

**Table 5:** Illustrative variables in the proposed Human–Skills–Equipment–Environment framework.

Domain	Symbol	Illustrative variable	Representative evidence
Human	$H_{1}$	Fatigue and exertion state	(4, 5)
Human	$H_{2}$	Physiological state and posture	(70)
Human	$H_{3}$	Position, velocity, and trajectory	(70, 109)
Skills	$S_{1}$	Technique and tactical strategy	(159)
Skills	$S_{2}$	Speed and execution during turns	(22, 23)
Equipment	$Eq_{1}$	Helmet, suit, and impact protection	(66, 110)
Equipment	$Eq_{2}$	Wearable and embedded sensing	(30, 59)
Equipment	$Eq_{3}$	Binding release and active protection	(15, 54)
Equipment	$Eq_{4}$	Ski geometry and sidecut	(1, 19)
Equipment	$Eq_{5}$	Ski and boot deformation or stiffness	(26, 34, 35)
Environment	$En_{1}$	Course infrastructure and barriers	(22, 110)
Environment	$En_{2}$	Course geometry and terrain	(22, 23)
Environment	$En_{3}$	Impact systems and safety nets	(66, 110)
Environment	$En_{4}$	Gates and gate contact	(66, 159)
Environment	$En_{5}$	Snow state and ski–snow interaction	(116, 117)

Inclusion indicates relevance to a future measurement framework, not proven causal contribution or validated weighting.

### 7.3 A conceptual skier safety index

A future study could investigate whether the four domains can be combined into a calibrated index. Let $x_{\mathrm{kj}}\in [0,1]$ denote a normalised variable j within domain k, and let $a_{\mathrm{kj}}$ be a within-domain weight. A domain score could be written as

$$F_{k}=\frac{\sum_{j}a_{\mathrm{kj}}x_{\mathrm{kj}}}{\sum_{j}a_{\mathrm{kj}}},\;k\in \{H,S,Eq,En\},$$ (1)

and a conceptual Skier Safety Index (SSI) as

$$\mathrm{SSI}=100\;\frac{w_{H}F_{H}+w_{S}F_{S}+w_{Eq}F_{Eq}+w_{En}F_{En}}{w_{H}+w_{S}+w_{Eq}+w_{En}}.$$ (2)

Equations 1, 2 are definitions of a possible model class, not an operational formula. No values are assigned to $x_{\mathrm{kj}}$, $a_{\mathrm{kj}}$, or $w_{k}$, and no safety threshold is proposed. Equal within-domain weighting would correspond to setting all $a_{\mathrm{kj}}=1$, but there is currently no evidence that equal weighting is appropriate.

The formulation has major limitations: it assumes additivity and, unless interaction terms are introduced, ignores dependencies among domains; normalisation is population- and context-dependent; missing or noisy measurements may bias the score; the outcome to be predicted must be defined separately (fall, injury, or injury severity); weights may vary by discipline and skier population; and a high or low value would not imply causation. Calibration, sensitivity analysis, uncertainty quantification, internal validation, and external prospective validation would all be required before the SSI could be interpreted. Until then, the index should be regarded only as a transparent hypothesis for organising future data collection.

## 8 Conclusions

Smart equipment, sensing, digital twins, and multibody simulation provide complementary capabilities, but the evidence does not support treating them as equally mature injury-prevention technologies. Conventional release bindings and protective equipment already perform direct safety functions. Wearable, video, GNSS, and force-measurement systems are increasingly capable of measuring skier behaviour and loading, yet prospective evidence that they reduce injuries remains limited. Predictive digital twins, adaptive bindings, robotic intervention, and AI-guided decisions are still experimental or conceptual and depend on rigorous validation.

The most consequential research need is therefore a validated measurement and modelling pipeline: standardised multimodal data, transparent model verification, independent human-subject validation, uncertainty reporting, and prospective evaluation of false alarms and missed events. The proposed H–S–Eq–En taxonomy and SSI are offered only as testable organising hypotheses for this work. Their value will depend entirely on whether future studies can calibrate and validate them across skiers, disciplines, equipment, and snow conditions.
